# Reliability and validity of the Persian version of 5-D itching scale among patients with chronic kidney disease

**DOI:** 10.1186/s12882-020-02220-x

**Published:** 2021-01-07

**Authors:** Amin Kordi Yoosefinejad, Fatemeh Karjalian, Marzieh Momennasab, Shahrokh Ezzatzadegan Jahromi

**Affiliations:** 1 Physical therapy Department, School of Rehabilitation Sciences, Shiraz University of Medical Sciences, Shiraz, Iran; 2 Student Research Committee, School of Nursing and Midwifery, Shiraz University of Medical Sciences, Shiraz, Iran; 3 Department of Nursing, School of Nursing and Midwifery, Shiraz University of Medical Sciences, Zand St., Namazee Sq, Shiraz, 7193613119 Iran; 4 Nephro-urology research center, Shiraz University of Medical Sciences, Shiraz, Iran

**Keywords:** 5-D pruritus scale, Validity, Reliability, Hemodialysis, Chronic kidney disease

## Abstract

**Background:**

Hemodialysis is considered a major therapeutic method for patients with chronic kidney disease. Pruritus is a common complaint of hemodialysis patients. The 5-D pruritus scale is amongst the most common tools to evaluate several dimensions of itch. Psychometric properties of the 5-D scale have not been evaluated in Persian speaking population with hemodialysis; hence, the objective of this study was to assess reliability and validity of the Persian version of the scale.

**Methods:**

Ninety hemodialysis patients (men: 50, women: 40, mean age: 54.4 years) participated in this cross-sectional study. The final Persian version of 5-D scale was given to the participants.

Tests Compared: One-third of the participants completed the scale twice within 3–7 days apart to evaluate test- retest reliability. Other psychometric properties including internal consistency, absolute reliability, convergent, discriminative and construct validity, floor/ceiling effects were also evaluated.

**Results:**

The Persian 5-D scale has strong test-retest reliability (ICC= 0.98) and internal consistency (Cronbach’s alpha= 0.99). Standard error of measurement and minimal detectable change were 0.33 and 0.91, respectively. Regarding convergent validity, the scale had moderate correlation with numeric rating scale (*r* =0.67) and quality of life questionnaire related to itch (*r* = 0.59). Exploratory factor analysis revealed two factors within the scale. No floor or ceiling effect was found for the scale.

**Conclusion:**

The Persian version of 5-D the itching scale is a brief instrument with acceptable reliability and validity. Therefore, the scale could be used by experts, nurses, and other health service providers to evaluate pruritus among Persian speaking hemodialysis patients.

**Supplementary Information:**

The online version contains supplementary material available at 10.1186/s12882-020-02220-x.

## Background

Chronic kidney disease (CKD) is a public health problem across the word. Hemodialysis is a major therapeutic method for many patients with CKD especially for those at end-stage renal disease with a prevalence of 22 to 84% [1]. Pruritus is a common complication among hemodialysis patients [2]. Pruritus is an unpleasant sensation, accompanied by desire to scratch the affected area [1] affecting up to 46% of hemodialysis patients [3]. The prevalence of pruritus among Iranian hemodialysis patients was reported as 41.9 to 48.3% [4]. Not only does the pruritus impact quality of life in hemodialysis patients, but also it has high psychological burdens [5]. Several scales have been developed to quantify the rate of pruritus. The Visual Analogue Scale (VAS), the Eppendorf Itch Questionnaire [6], the Skindex [7], the Itch medical outcome study [8], and the 5-D pruritus scale are among the most popular tools used to evaluate the rate of itching. Currently, there is no consensus on a universal scale which would be able to evaluate severity, degree, duration, and impact on quality of life among hemodialysis patients.

The 5-D itch scale was first developed by Elman et al. as a new measure of itching [9]. It was validated with a numerical rating scale (NRS) in patients with human immunodeficiency virus, skin, liver or kidney disease [9]. In contrast to unidirectional scales such as NRS and VAS, the 5-D pruritus scale is a multidimensional measure. Previously 5-D itch scale was used as an assessment tool to evaluate multidimensional aspects of pruritus among hemodialysis patients [10]. Considering that pruritus as one of the most common and frustrating symptoms among hemodialysis patients, evaluating different dimensions of itching seems mandatory. Moreover, there is lack of an agreed-upon valid multidimensional scale for Persian-speaking experts and health care providers to evaluate pruritus among hemodialysis patients. Therefore, the objective of this study was to evaluate the validity and reliability of the Persian version of 5-D pruritus scale.

## Methods

This cross-sectional study was part of a more extensive study conducted between August 2018 and February 2019 at dialysis centers affiliated with Shiraz University of Medical Sciences, Shiraz, Iran. The study was approved by the Ethics Committee of the Vice Chancellery of Shiraz University of Medical Sciences in concordance with the standards of Helsinki declaration (Ethics number: IR.SUMS.REC.1396.2.9). Ninety patients were recruited from dialysis centers affiliated with Shiraz University of Medical Sciences, Shiraz, Iran. Patients aged at least 18 years old and received hemodialysis for more than 3 months were eligible to participate in the study. Patients with autoimmune diseases such as systemic lupus erythematous, and those with liver complications were excluded. The sample size was calculated as a minimum of 50 patients based on the ratio between the number of items in scale to participants which is considered as a ratio of 1:10 [11]. All patients signed written informed consent form before participating in the study.

Five dimensions of itching beginning with letter “D” are included in 5-D pruritus scale and are evaluated during the previous 2 weeks. These dimensions include duration; degree; direction; disability; and distribution of itching. The first three items (duration, degree, direction) are single-item domains and are scored from 1 (‘less involvement according to the item’) to 5 (‘most involvement according to the item’). Disability represents a multiple-item domain and includes the effects of itching on daily activities such as sleep, leisure/social activities, housework/errands and work/school. The score for the disability domain is obtained by obtaining the highest score within four evaluated sub domains. The fifth item (Distribution) evaluates the presence of itching within 16 body parts over the previous 2 weeks. Regarding the number of affected parts, five scoring bins are constructed. The sum of 0–2 is considered as the score of bin 1, sum of 3–5= score of 2, sum of 6–10 =score of 3, sum of 11–13= score of 4 and sum of 14–16=score of 5. 5-D scores range from 5 (no pruritus) to 25 (most severe pruritus).

### Translation process

To translate the 5-D itching scale for Persian-speaking dialysis patients, we followed the guidelines served as a template for the translation and cross-cultural adaptation in medical literature introduced by Beaton et al. [12].

Initial translation: The English version of the 5-D scale was translated into Persian by two native bilingual translators independently. One translator was an experienced nurse who was aware of the concept of the scale. Another was neither medical staff nor informed of the concepts of the scale.

Synthesis of the translations: Translators and a recording observer synthesized the initial results. Comparing the original scale with translations, a written report documenting the synthesis process was provided. Hence, a finalized forward translation of 5-D scale was provided.

Back translation: forward translation of the 5-D scale was given to two bilingual translators whose native language was English and were not medical staff. The translators were not aware of the concepts of scale.

Expert committee: The aim of this stage was to consolidate all the versions and to develop pre-final version of scale. Decisions were made on semantic, idiomatic, experiential, and conceptual equivalences. The committee comprised of the translators, two expert nurses, a health professional and a methodologist.

Test of the pre-final version: Eight hemodialysis patients with itching completed the scale and had a cognitive interview about the concept of items and the provided responses. l Items were easily comprehended by interviewees.

Submission of documentation to the coordinating committee for appraisal of the adaptation process: Expert committee came to a consensus on the final adapted version of scale.

### Reliability

Relative reliability of the 5-D scale was evaluated with the test-retest intraclass correlation coefficient (ICC) and internal consistency of the scale. Thirty patients completed the 5-D scale twice, 3 to 7 days apart. Internal consistency was evaluated with Cronbach’s alpha.

Absolute reliability of the scale was evaluated with calculating standard error of measurement (SEM) and minimal detectable change (MDC).

SEM is calculated as follows:

$$ \mathrm{SEM}=\mathrm{SD}\times \surd 1-\mathrm{ICC} $$

“SD” stands for standard deviation and ICC stands for intraclass correlation coefficient. MDC is calculated employing the formula: MDC= 1.96 × √ 2 × SEM [13].

### Validity

Convergent validity of the 5-D scale was evaluated with a numeric rating scale (NRS) and scales. The NRS is a uni-dimensional scale comprised of 11 points. Achieving zero points is interpreted as “no pruritus”,1–3 points on NRS is interpreted as mild itch, 4–6 points as moderate itch, 7–8 points as severe and 9 points or more points is interpreted as severe pruritus [14]. NRS ≥ 4 was considered as symptomatic pruritus [14].

The Itchy QoL questionnaire is a standard, common self-reported instrument designed to evaluate quality of life among patients with pruritus. It was first developed by Desai et al. (2008) and consists of 26 questions evaluating three different major aspects of the quality of life in patients with pruritus [15]. The first aspect is about symptoms experienced by the individual and includes the first six questions. Questions 7 to 16 evaluate “functioning” dimension and questions 17 to 26 evaluate the “emotion” dimension of the individuals. Each question is designed as a Likert type item from 1 (never) to 5 (always). Scores are considered separately within each dimension and also totally as the aggregate score of itchy QoL questionnaire. Score range is 26 to 130; the higher the score the greater the quality of life. Psychometric properties of the Persian version of Itchy QoL were evaluated by Tari et al. [16]. Construct validity of the scale was evaluated with exploratory factor analysis using principal component analysis and varimax rotation [17]. The Eigenvalues greater than one were considered as having a significant contribution in explaining the overall model variation. Sampling adequacy was examined using Bartlett’s test of sphericity and Kaiser–Mayer–Olkin (KMO). A KMO value greater than 0.6 was considered acceptable, and 0.80 indicates a good level of compatibility of the variables within the test.

To determine discriminative validity, the Mann-Whitney U test was used to evaluate the scores of the participants at baseline and re-test to observe no significant difference between the results [18].

Floor and ceiling effects were calculated for the Persian version of 5-D scale. These are defined as if more than 15% of the participants achieve either the least (floor) or the greatest (ceiling) scores.

## Results

**Fig. 1:**
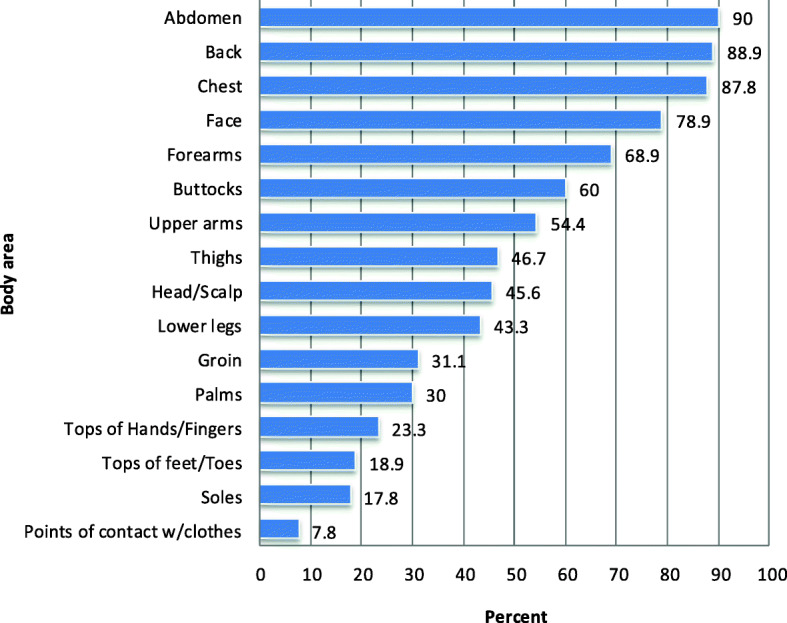
Distribution of itch percentage among different regions of body in patients receiving hemodialysis

A total of 90 patients undergoing hemodialysis was recruited. Demographic characteristics of the participants are summarized in Table 1. The mean score of the Persian version of the 5-D scale was 17.69 ± 3.03 with scores ranging from 11 to 25. The mean time for receiving hemodialysis was 7.04 ± 3.01 years (Range: 13 years) and the mean itching duration was 21.39 ± 9.20 months (range: 41 months). Scoring as per the domains mentioned in the Persian version of the 5-D sitching scale is shown in Table 2. Regarding the NRS grading system for itch, 11 (12.2%) of the participants had moderate degree of itching, 50 (55.6%) of the individuals had severe itching and 29 (32.2%) of the participants had very severe itching. It means all the participants had symptomatic pruritus (NRS≥ 4). Considering the distribution of itch, the abdomen had the most considerable percentage of itch (90%) while contact with clothes had the least percentage (7.8%). Distribution percentage of various parts of body is exhibited in Fig. 1.

**Table 1:** Demographic data of the participants (*n*=90)

Demographics	n (%)
**Sex**	
**Men**	50 (55.6)
**Women**	40 (44.4)
**Age (years)**	
**Range**	39
**mean**	54.40
**Median**	58
**Education**	
**Illiterate**	50 (55.6)
**primary level (6 years of education)**	6 (6.7)
**secondary level (12 years of education)**	33 (36.7)
**tertiary level (≥ 16 years of education)**	1 (1.1)
**Marital status**	
**Married**	75 (83.3)
**Single**	7 (7.8)
**Widowed**	4 (4.4)
**Divorced**	4 (4.4)
**Settlement**	
**Urban**	24 (26.7)
**Suburb**	66 (73.3)
**Expenses for treatment borne by**	
**Government**	42 (46.7)
**Family**	48 (53.4)

**Table 2:** Scoring as per the domains mentioned in the Persian version of 5-D itching scale

Variable	N (%)
Duration (Mean score =3.29 ± 0.98)	
Less than 6 h a day	3 (3.3)
6–12 h a day	12 (13.3)
12–18 h a day	44 (48.9)
18–23 h a day	18 (20)
All day	13 (14.4)
Degree (Mean score= 4.18 ± 0.59)	
Not present	0 (0)
Mild	0 (0)
Moderate	9 (10)
Severe	55 (61.1)
Unbearable	26 (28.9)
Direction (Mean score=3.63± 0.66)	
Completely resolved	8 (8.9)
Much better, but itching is still present	42 (46.7)
Little bit better but still presents	39 (43.3)
Unchanged	1 (1.1)
Getting worse	0 (0)
Disability (Mean score=9.05 ± 2.79)[Minimum= 4, Maximum= 16]	
Sleep (Mean score= 3.51 ± 1.25)	
Never affects sleep	8 (8.9)
Occasional delays falling asleep	13 (14.4)
Frequent delays falling asleep	16 (17.8)
Delays falling asleep and occasionally wakes me up at night	31 (34.4)
Delays falling asleep and frequently wakes me up at night	22 (24.4)
Leisure/social activity (Mean score= 2.11± 0.77)	
Never affects leisure/social activity	20 (22.2)
Rarely affects leisure/social	42 (46.7)
Occasionally affects leisure/social	26 (28.9)
Frequently affects leisure/social	2 (2.2)
Always affects leisure/social	0 (0)
Housework/errands (Mean score = 1.72 ± 0.72)	
Never affects house work/errands	38 (42.2)
Rarely affects house work/errands	40 (44.4)
Occasionally affects house work/errands	11 (12.2)
Frequently affects house work/errands	1 (1.1)
Always affects house work/errands	0 (0)
Work/School (Mean score= 1.71 ± 0.76)	
Not applicable	40 (44.4)
Never affects Work/School	1 (1.1)
Rarely affectsWork/School	36 (40)
Occasionally affectsWork/School	11 (12.2)
Frequently affectsWork/School	2 (2.2)
Always affectsWork/School	0 (0)
Distribution (Mean score= 3.00 ± 0.75) [Minimum=1, Maximum=5]	
Scoring Bin 1	1 (1.1)
Scoring Bin 2	19 (21.1)
Scoring Bin 3	52 (57.8)
Scoring Bin 4	15 (16.7)
Scoring Bin 5	3 (3.3)
Total score (Mean score = 17.69 ± 3.03)	
6–10	0 (0)
11–19	62 (68.9)
20–25	28 (31.1)

### Reliability

ICC between the test and retest of the Persian version of the 5-D scale was 0.98 (95% confidence interval: 0.96–0.99) and the overall Cronbach’s alpha of the scale was 0.99. The SEM and MDC of the Persian version of the scale were calculated as 0.33 and 0.91 respectively. Further reliability measures are shown in Table 3.

**Table 3:** Reliability measures of the Persian version of 5-D scale

Domains	Test Median	Retest Median	Corrected item total correlation	Cronbach’s alpha if item deleted	ICC
Duration	3	3	0.62	0.85	0.98
Degree	2	2	0.60	0.85	0.99
Direction	3	3	0.47	0.86	0.99
Disability					
Sleep	2	2	0.70	0.85	0.97
Leisure/Social life	4	4	0.68	0.84	0.98
House work	4	4	0.65	0.85	0.99
Work/School	4	4	0.61	0.85	0.99
Distribution	2	3	0.48	0.86	0.80

### Validity

The Pearson correlation coefficient between the Persian version of the 5-D scale and the NRS was moderate (*r*=0.67, *p*< 0.001). Also, the Persian 5-D had a moderate degree of correlation with Itchy-QoL questionnaire (*r*= 0.59, *p*< 0.001).

**Fig. 2:**
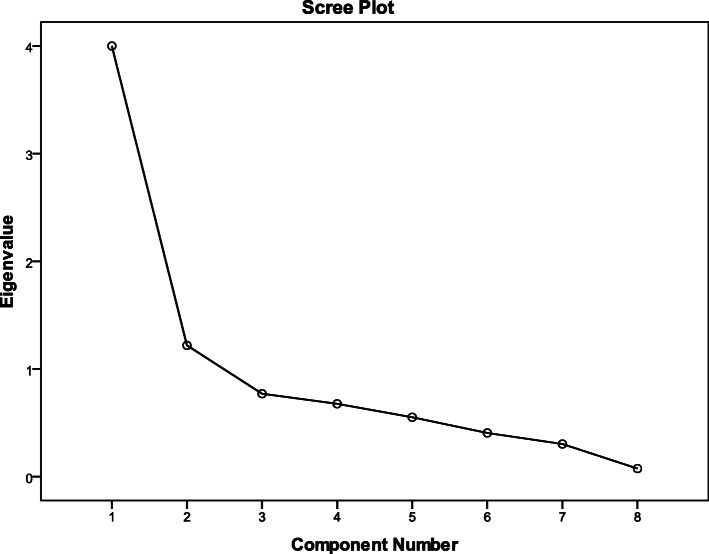
Scree plot of exploratory factor analysis of the Persian version of the 5-D scale

Exploratory factor analysis revealed the Persian version of the 5-D scale had two factors. One factor included duration, degree, direction, and sleep items while the other factor comprised of social, housework, school/work and distribution items. These factors explained 65.22% of the total observed variance. [KMO = 0.78, Bartlett’s test of sphericity was significant. (Chi-square= 371.60, *df=28*, *p*< 0,001)]. The scree plot of the exploratory factor analysis is depicted in Fig. 2. The scree plot is a commonly used graphical method for determining the number of the components to extract.

As can be observed from Fig. 2, two factors (x-axis) could be extracted from the Persian version of the 5-D itching scale with Eigenvalues greater than one (y-axis) [19].

Discriminative validity revealed no significant difference between the achieved scores of the dimensions of the 5-D itching score at baseline (*n*=90) and re-test (*n*=30). The comparison of the dimensions between test and re-test is summarized in Table 4.

**Table 4:** Comparison of the scores of the dimensions of the Persian version of 5-D itching scale

Domains	Test (n=90)		Re-test (n=30)		Mann-Whitney U ***p***-value
	median	IQR (25–75%)	median	IQR*(25–75%)	
**Duration**	3	1–4	3	1–4	**> 0.99**
**Degree**	2	1–2	2	1–2	**> 0.99**
**Direction**	3	2–3	3	2–3	**> 0.99**
**Disability**					
**Sleep**	2	1–4	2	1–4	**0.32**
**Leisure/Social life**	4	3–5	4	3–5	**> 0.99**
**House work**	4	4–5	4	4–5	**> 0.99**
**Work/School**	4	4–5	4	4–5	**> 0.99**
**Distribution**	2	2–3	3	2–3	**0.06**

*: Inter quartile range

Considering the least (11, 2.2%) and the highest (25, 1.1%) achieved scores of the Persian version of the 5-D scale, neither a floor nor ceiling effect was observed in the scale.

## Discussion

The 5-D itching scale is a commonly used patient reported outcome measure in pruritus which was validated into six different languages. The aim of this study was to assess the reliability and validity of the Persian version of the 5-D itching scale among Persian-speaking patients receiving hemodialysis.

The Persian version of the 5-D itch scale revealed excellent internal consistency (Cronbach’s alpha = 0.99) and excellent agreement for test-retest reliability (ICC= 0.98). Compared with the Indonesian [20] (Cronbach’s alpha= 0.67), Malaysian (Cronbach’s alpha= 0.86) [21], Urdu version [1] (Cronbach’s alpha= 0.91), and Arabic version [22] (Cronbach’s alpha= 0.85) of the 5-D itch scale, the Persian version showed a higher internal consistency. The original version of the scale reported the internal consistency as 0.73 [9]. The Japanese version of the scale did not report the internal consistency [23]. In line with Urdu (ICC= 0.91), Arabic (ICC=0.85), and the original version (ICC=0.96), the Persian version of the 5-D scale showed excellent test-retest reliability. We also evaluated the absolute reliability of the Persian version with calculating SEM and MDC. To our best knowledge, no other version of the scale has evaluated the absolute reliability; so, our findings could not be compared to other versions of the scale.

The Persian version of the 5-D scale had a moderate degree of correlation with both NRS (*r*=0.67) and the Persian version of Itchy QoL questionnaires (*r*=0.59). The original version of the scale also showed a moderate correlation (r=0.69) with the VAS. The Indonesian version had a strong correlation with the dermatology life quality index and the VAS [20]. The Japanese version of the scale showed strong correlation with vas (*r*=0.70), daytime itchiness (*r*=0.64), and nighttime itchiness (*r*=0.63) [23]. Other versions of the scale did not evaluate the correlation of the scale with other related instruments. Among the pruritus scales, the Itchy QoL was the only scale to be validated into Persian; hence, we evaluated the convergent validity of the Persian version of the 5-D scale with the above-mentioned scales. No other version evaluated the correlation of the 5-D scale with the Itchy QoL.

Exploratory factor analysis revealed two factors for the Persian version of scale. The first factor was “daily routine activities” and the other factor was “pattern of itching”. This was in line with the Malaysian version of the scale [21]. The Malaysian version also revealed two factors with similar components for each factor. Moreover, the Urdu version of the scale revealed two factors; however, the loading components were different from those obtained with the Persian version of scale [1]. Although the Arabic version of the scale claimed to have evaluated the factor analysis of the scale, only the values of KMO and Bartlett’s test of sphericity were reported and no factor rotation was carried out [22]. The factor analysis was not performed on the other versions of scale. Discriminative validity of the scale revealed stability of the scale as the participants interpreted items similarly at baseline and retest. To the best of our knowledge, only the Urdu version of the scale evaluated discriminative validity and their results are congruent with ours [1]. The Persian version of the scale revealed either no floor or ceiling effects; however, we could not compare our findings due to dearth of evidence.

To our best knowledge, our study was the first to evaluate absolute reliability and floor/ceiling effects of the 5-D itching scale. Our study had some limitations. We did not evaluate the responsiveness of the scale; thus, future studies are warranted to assess the responsiveness of the scale following several invasive or non-invasive therapies. Also, because of the lack of adequate number of Persian-validated pruritus scales, we performed convergent validity with just the Itchy QoL. Considering our study, future studies have more available instruments to evaluate validity of their tools.

## Conclusion

The Persian version of the 5-D itching scale is a brief tool with acceptable reliability and validity. Therefore, experts, nurses, and other health service providers can use it to evaluate pruritus among Persian speaking patients with CKD.

## Supplementary Information

**Additional file 1.**

## Data Availability

All data generated or analysed during this study are included in this published article [and its supplementary information files (additional file 1)].

## Abbreviations

CKD: Chronic kidney disease
VAS: Visual analogue scale
NRS: Numerical rating scale
ICC: Intraclass correlation coefficient
SEM: Standard error of measurement
MDC: Minimal detectable change
SD: Standard deviation
QoL: Quality of life
KMO: Kaiser–Mayer–Olkin
